# An Atypical Case of Thoracic Endometriosis Syndrome Mimicking Pulmonary Tuberculosis

**DOI:** 10.1155/crra/1708094

**Published:** 2025-05-20

**Authors:** Victor Mhezi, Zuhura Nkrumbih, Magafu Majura

**Affiliations:** ^1^Department of Radiology and Imaging, Songea Regional Referral Hospital, Songea, Ruvuma, Tanzania; ^2^Department of Radiology and Imaging, Muhimbili University of Health and Allied Sciences, Dar-es-Salaam, Tanzania; ^3^Department of Obstetrics and Gynecology, Songea Regional Referral Hospital, Songea, Ruvuma, Tanzania

**Keywords:** catamenial, endometrioma, hemoptysis, hemothorax, hydropneumothorax, pneumothorax, pulmonary tuberculosis, thoracic endometriosis syndrome

## Abstract

**Background:** Thoracic endometriosis syndrome (TES) is a rare form of endometriosis characterized by the presence of functioning endometrial tissue in the thoracic cavity. Patients are women of reproductive age, with a genetic link as a significant risk factor. Patients present with long-standing chest symptoms and signs that mimic pulmonary tuberculosis (PTB). The crucial issue for establishing the diagnosis is the cyclicity of signs and symptoms which occur along with the menstrual cycle.

**Clinical Case:** A 34-year-old businesswoman had recurrent pelvic pain and heavy menses for 6 years, recurrent chest pain for 5 years, and recently coughing blood for 3 days. Symptoms peaked during menstruation. She reported a maternal grandmother with similar symptomatology. For the past 6 years, she was treated for recurrent pneumonia and PTB without improvement. Examination revealed right-sided pleural effusion and generalized pelvic tenderness. The catamenial nature of her symptoms led to a suspicion of TES, with PTB. Pleural fluid analysis showed exudative effusion, and Gene X-pert for MTB was negative. CA-125 was elevated, a nonspecific endometriosis marker. Pelvic ultrasound revealed features of pelvic endometriomas. Serial chest X-ray and CT scan showed right hydropneumothorax, lung mass, lung collapse, and pulmonary fibrosis. Multiple chest tubes were placed for the recurrent hydropneumothorax management. Exploratory laparotomy with bilateral ovarian cystectomy was done, and histology revealed ovarian hemorrhagic cysts and salpingitis. Hormonal suppression initiated as mainstay of treatment. She is monitored monthly as an outpatient to assess treatment efficacy and condition progression.

**Conclusion/Learning Points:** TES is a form of endometriosis involving the thoracic cavity, affecting women of reproductive age. TES may mimic PTB but symptoms correlate with the menstrual cycle (catamenial in nature). In Tanzania, diagnostic challenges persist due to its nonspecific symptoms, inadequate clinicians' awareness, and lack of treatment guideline national wide.

## 1. Introduction

Endometriosis is a chronic gynaecological condition defined as the presence of functional endometrial glands and stroma-like lesions outside the uterus. Typically, it presents in young women of reproductive age years; however, postmenopausal women are diagnosed with very few instances [1].

The most common site for endometriosis is in the ovaries and pelvic peritoneum. Other locations include caesarean section scars (scar endometriosis), deep subperitoneal, gastrointestinal and urinary tracts, or subcutaneous tissues [1]. Extra-abdominal location of endometriosis which includes the chest, also termed as “thoracic endometriosis syndrome” (TES) is uncommon, usually diagnosed in the setting of long-standing (> 5 years) pelvic endometriosis [2].

The exact cause of TES is unclear, but several theories have been made. The “metastatic theory” involves retrograde menstruation, vascular or lymphatic spread, or iatrogenic implantation to the chest [3]. The “metaplastic theory” suggests the metaplasia Müllerian remnant [4]. Lastly the “induction theory” suggests that chemical mediators from shed endometrial tissue trigger ectopic tissue formation [5].

TES usually presents with catamenial (cyclic) pleuritic chest pain, hemoptysis, pneumothorax, or pleural effusions, and often in the absence of symptoms associated with pelvic endometriosis. The key clinical indicator is the cyclical nature of signs and symptoms, which align with the menstrual cycle [6–8].

Diagnosis of TES is challenging as the condition is rarely identified as the cause of pneumothorax or hemothorax by chest imaging using an X-ray or computed tomography (CT) [9]. Cytology can occasionally detect endometrial epithelial cells following thoracentesis of a hemothorax or biopsy of suspicious areas, with limited sensitivity [10]. Endoscopy and video-assisted thoracoscopic surgery may confirm the diagnosis [11]. However, TES is primarily a diagnosis of exclusion, determined based on clinical correlation to menstrual cycles, as neither chest imaging nor endoscopy provide definitive diagnosis. [12].

The significance of this case lies in the diagnostic challenge of TES, which was initially mistaken for pulmonary tuberculosis (PTB), a common and prevalent respiratory disease in developing countries. It highlights the necessity of clinical suspicion of TES in women of reproductive age with recurrent thoracic symptoms and inconclusive PTB evaluations, promoting awareness to clinicians on diagnosis and management of this rare condition.

## 2. Case Presentation

### 2.1. Clinical History

A 34-year-old female, a businesswoman, presented with chief complaints of recurrent lower abdominal pain for 6 years, recurrent right-sided chest pain for 5 years, and coughing blood for 3 days.

Recurrent lower abdominal pain, worsened during the first 3 days of menstruation, accompanied by heavy, prolonged menstrual periods, painful intercourse, nausea, vomiting, and occasional nonbloody loose stools. Then, 6 years of recurrent chest pain worsened during menstruation, accompanied by nonproductive cough peaking 3 days before and subsiding 3 days after onset menses. There were no reported history of fevers, night sweats, weight loss, or smoking. During her most recent menstrual period, the patient experienced coughing up of blood for 3 days, which occurred 2–3 times a day and but resolved after menstruation.

After the onset of these symptoms, 6 years ago, the patient was diagnosed with PTB and she was kept on anti-TB regime over the course of 6 months in two different occasions without improvement. In the following years, she was kept on the management for recurrent pneumonias, again without improvement.

On gynaecological history, she is P1L1, attained menarche at 13 years, irregular menstrual cycles of 28 days, and flow of 7–9 days. No history of contraceptive use. She conceived at 16 years without any obstetric complication. For 6 years of this illness, she presented with secondary infertility. She reports to have maternal grandmother who had near similar symptomatology at her younger age. No reported history of surgeries or other chronic illness.

### 2.2. Physical Examination

The patient was clinically stable with stable vital signs. On respiratory examination, she presented with tactile vocal fremitus, stony dull percussion notes, decreased breath sounds, and decreased vocal resonance on the right infrascapular and interscapular regions, features suggestive of right-sided pleural effusion. Pelvic examination reveals mild generalized pelvic tenderness. Per vaginal examination was unremarkable. Other systemic examinations were essentially normal.

### 2.3. Diagnosis Formulation

Provisional diagnoses include TES and pelvic endometriosis, presenting with secondary infertility. Differential diagnoses include post TB sequelae to rule out extrapulmonary TB. Points against TB are catamenial nature of symptoms and absence of constitutional symptoms.

### 2.4. Imaging Findings

**Figure 1:**
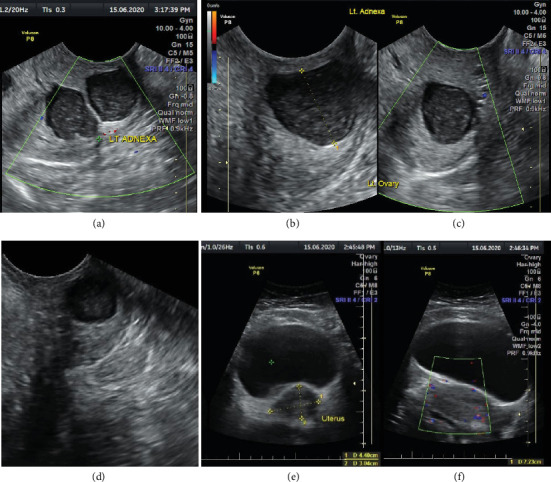
Pelvic ultrasound images (a–c) (left adnexa) and (d) (right adnexa): demonstrating multiple rounded and ovoid hypoechoic lesions some with septa. The largest lesion measured 1.52 × 1.5 cm (b). Lesions show homogeneous low-level internal echoes with posterior acoustic enhancement. No colour flow within lesions upon colour Doppler application, features suggestive of chocolate cysts. (e, f) The uterus in transverse and longitudinal views, with normal size, echogenicity, and normal myometrial vascularity.

**Figure 2:**
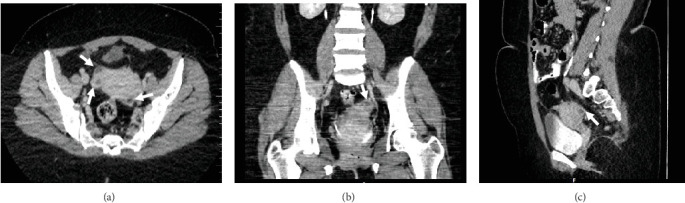
Contrasted abdomen-pelvic CT scan images (a) axial, (b) coronal, and (c) sagittal: demonstrating bilateral adnexal hemorrhagic cystic masses adjacent to the uterus with thick septation and walls enhancement (white arrows).

**Figure 3:**
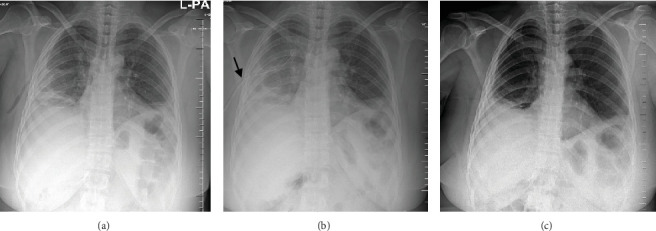
(a–c) Frontal CXR images each taken 1 month apart few days after onset of her menses: All demonstrate homogenous opacity involving the right mid and lower zones obscuring the right cardiac border, obliteration of both right costophrenic and cardiophrenic angles with positive meniscus sign, in keeping with recurrent catamenial right-sided pleural effusion. Black arrow (b) points a thoracentesis tube in the right hemothorax, which later it was repositioned.

**Figure 4:**
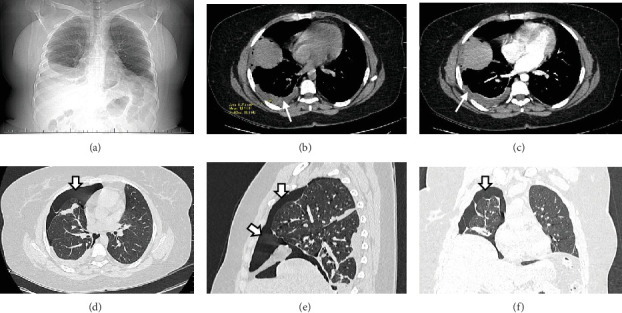
(a) Scanogram shows right-sided pleural effusion. (b) An axial chest CT scan, soft tissue window noncontrasted and (c) contrasted, showed free fluid collection in pleural space (14HU) involving the right hemithorax in keeping with right pleural effusion (long arrows). Additionally, there is a pleural-based nonenhancing hemorrhagic mass was observed within the middle lobe. (d–f) Lung windows showed peripheral air-dense region devoid of lung markings (short arrows) in the right hemithorax (pneumothorax) with underlying right lung collapse and areas pulmonary fibrotic changes.

A pelvic ultrasound scan revealed left multiple complex cystic adnexal masses with differential diagnoses of endometriomas/hemorrhagic cysts, as described in Figure 1. An abdomen–pelvic CT scan was done to rule out intra-abdominal manifestation of TB, which showed multiple small bilateral adnexal cystic lesions and pelvic adhesions (Figure 2). Serial chest X-ray (CXR) studies were taken each 1 month apart, which all revealed right-sided pleural effusion (Figure 3). High-resolution CT scan (HRCT) of the chest was requested for further evaluation of recurrent right pleural effusions (Figure 4), which revealed right hydropneumothorax, with underlying right lung collapse, right pulmonary fibrotic changes, and middle lobe plural-based hemorrhagic lung mass.

### 2.5. Laboratory Investigations

CA-125 was markedly raised to 114.4 units/mL (normal 0–35), a nonspecific marker for endometriosis. Erythrocyte sedimentation rate (ESR) was mildly raised. Bleeding indices, full blood count, blood chemistry, serology, and urine profile were within normal limits. Sputum for Gene X-pert was negative for mycobacterium TB (MTB). Cytology analysis of pleural fluid revealed exudative effusion with degenerative endometrial like tissues and inflammatory cells, with no growth on culture.

### 2.6. Final Diagnosis

Final diagnoses were reached to be TES and pelvic endometriosis presenting with secondary infertility. This is the due to catamenial nature of symptomatology, raised CA-125 levels (endometriosis marker), supportive serial imaging findings (pelvic endometriomas, right-sided hydropneumothorax, and pleura-based hemorrhagic mass), and cytology analysis of pleural fluid. PTB diagnosis was pointed-out due to the absence of constitutional symptoms like fever, night sweats and weight loss, negative Gene X-pert, and failure to respond to standard TB treatment.

### 2.7. Management

She had multiple chest tubes for management of recurrent hydropneumothorax. She also had exploratory laparotomy with findings of inflamed blocked fallopian tubes and hemorrhagic cysts were seen on both ovaries. Ovarian cystectomy and adhesionolysis were done. Ovarian and tubal biopsies were taken; histology results of a section of tissues revealed endometrial tissues-like inflammatory and hemorrhagic cells lined by residual cells, a conclusion of ovarian endometrioma/hemorrhagic cysts, and salpingitis.

She was kept under pain management and hormonal suppression therapy using gonadotropin hormone-releasing hormone (GnRH) agonist (Inj. Goserelin 3.6 mg s/c monthly). Possible in vitro fertilization (IVF) for treatment of secondary infertility was thought.

### 2.8. Follow-Up

The patient has a monthly follow-up as an outpatient in gynaecology and cardiothoracic surgical clinics for monitoring of treatment and condition progress. Significant improvement in clinical signs, symptoms, and improved imaging findings, which predict good prognostic outcomes and promising treatment response.

## 3. Discussion

TES is chronic gynaecological disorder that features the presence of functional endometrial tissues within the thoracic cavity, affecting the lung parenchyma, airways, diaphragm, or pleural surfaces. TES is rare but the most prevalent form of endometriosis found outside the pelvic and abdominal regions [13, 14]. TES typically presents at a mean age of 35 years (range 15–54 years) and more common in nulliparous women but can also see in women with previous childbirth history. The demographics of our 34-year-old index patient who is para 1, living 1, aligns with the common profile described in the literature and other similar reported cases [15, 16].

The exact cause of TES is unclear, but risk factors include previous surgeries, laparoscopic procedures, short menstrual cycles, and familial genetic predisposition. Our patient, lacking surgical or menstrual risk factors, has a maternal grandmother who had similar symptoms during her reproductive age. This finding aligns with the evidence supporting a hereditary component linked to TES [1, 15].

TES commonly manifests with catamenial chest pain, dyspnoea, hemoptysis, pneumothorax, hemothorax, and pulmonary nodules, with pneumothorax being the most frequent finding. The right hemithorax is involved in over 90% of cases. Our index patient exhibited recurrent hemoptysis, pneumothorax, pleural effusion, a middle lobe subpleural lung mass, and fibrotic changes in the right lung. While the right-sided predominance aligns with literature, the pulmonary fibrosis and lung mass are less commonly reported findings [14–17].

Additionally, this patient was diagnosed with TES alongside pelvic endometriosis and secondary infertility. The literature indicates a strong association of these three conditions. Up to 84% of women with pelvic endometriosis also have TES. This highlights the importance of thorough evaluation for patients with pelvic endometriosis to rule-out possible thoracic involvement [15, 18–20].

TES is a diagnosis of exclusion, with patient clinical presentation closely tied to the menstrual cycle, which is a key diagnostic hallmark. Serum CA-125 (a nonspecific laboratory marker for endometriosis), chest imaging, endoscopy, and histopathological assessment are crucial to support the diagnosis [11, 16, 21, 22]. Our patient had markedly elevated CA-125 levels, supported by chest X-ray and CT scan imaging findings. Pleural fluid cytology and ovarian and fallopian tube biopsies suggested features of endometriosis. Thoracic endoscopy was in our wishlist; however, it was not done, highlighting the limitation of the procedure in our clinical setup.

Diagnosing TES can be difficult as it often mimics other pulmonary conditions, with symptoms that may not always align with the menstrual cycle. This leads to delayed diagnoses, with many cases misclassified as spontaneous pneumothorax, especially when TES presents with pneumothorax. It can take 8–16 months for a conclusive diagnosis, typically after multiple symptom recurrences [10, 22].

In our case, the patient was initially managed as a case of recurrent pneumonia and PTB for almost 6 years, which were then ruled-out. PTB was strongly suspected due to the patient's persistent chest symptoms, including hemoptysis and CXR findings such as pleural effusion and particularly due to high incidence of PTB in Tanzania. This closely mirrors the challenges described in the literature, where TES is often underreported and misdiagnosed.

TES treatment primarily involves hormonal suppression therapy to induce regression of endometrial implants. This includes androgens, GnRH agonists, and progestogens [16, 23, 24]. This patient responded well to a goserelin injections (GnRH agonists therapy), with notable progress and improvement during follow-ups. Also, as observed in our case, nonsteroidal anti-inflammatory medications are particularly effective in pain management [16, 25].

Before a definitive diagnosis of TES was established, managing pleural effusion and pneumothorax using chest tube drainage alone was found ineffective, leading to recurrences with only transient improvement on CXRs. Similar findings have been reported in literature and perhaps additional chemical pleurodesis could have prevented these recurrences [10, 23, 26, 27].

Over 60% of reported TES cases, treatment involves combination of both medical therapy and minimally invasive techniques like thoracoscopic surgery; these procedures were unavailable in our set up. Additionally, if medical therapy fails and severe or recurrent TES, invasive segmentectomy or lobectomy is the preferred treatment, though relapse rates are high [11, 16, 23, 28].

## 4. Conclusion and Learning Points

TES diagnosis requires a multidisciplinary approach, including careful gynaecologic evaluation and assessment of cyclic pulmonary symptoms and laboratory, imaging, and endoscopy findings. But diagnosis is on clinical grounds, as imaging findings or endoscopy findings are nonspecific. In Tanzania, TES diagnosis remains challenging, mistaken for conditions like PTB. This is contributed by nonspecific disease presentation, inadequate diagnostic methods, limited clinicians' knowledge and awareness of disease, and the absence of standardized management guidelines at national or global levels.

## Data Availability

The data that support the findings of this study are openly available upon request.
